# The effect of the buteyko breathing technique on fatigue, exercise capacity, and asthma control in patients with asthma: a randomized controlled trial

**DOI:** 10.1007/s11845-026-04382-3

**Published:** 2026-04-11

**Authors:** Adile Neşe, Fatoş İpekçioğlu

**Affiliations:** 1 Department of İnternal Medicine Nursing, Vocational School of Health Services, Gaziantep University, Gaziantep, Turkey; 2 Bilen Barlık Dental Clinic, Gaziantep, Turkey

**Keywords:** Buteyko breathing technique, Asthma, Exercise capacity, Fatigue, Asthma control

## Abstract

**Objective:**

This study aimed to evaluate the effects of the buteyko breathing technique on fatigue, exercise capacity, and asthma control in patients with asthma.

**Methods:**

This randomized controlled pretest–posttest study was conducted between October 2024 and June 2025 in the outpatient clinic of a university hospital’s Department of Pulmonary Diseases. Ninety patients with asthma were randomly assigned to an intervention group (*n* = 45) or a control group (*n* = 45); the study was completed with 85 participants after five withdrawals. The intervention group received training and exercises based on the Buteyko breathing technique, while the control group continued routine medical treatment. Data were collected using the six-minute walk test, pulmonary function test parameters, the Fatigue Scale, and the Asthma Control Test.

**Results:**

Baseline measurements showed no significant differences between groups (*p* > 0.05). After the intervention, the six-minute walk distance increased significantly in the intervention group (*p* = 0.010), along with improvements in forced expiratory volume in one second (FEV₁) and the FEV₁/FVC ratio (*p* < 0.05). Fatigue scores decreased significantly, and asthma control test scores increased markedly in the intervention group (*p* = 0.001). No significant changes were observed in the control group, and no adverse events were reported.

**Conclusion:**

The buteyko breathing technique improves exercise capacity, reduces fatigue, and enhances asthma control in patients with asthma. As a safe and low-cost intervention, it may be considered a useful adjunct to standard asthma management.

**Reporting method:**

This randomised controlled trial used the CONSORT guidelines.

**Patient or public contribution:**

No Patient or Public Contribution.

## Introduction

Asthma, a chronic inflammatory airway disease, is a major public health problem affecting individuals of all age groups in many countries. While asthma may present as a mild condition that does not limit daily activities, it can also significantly impair both routine and basic functional activities, leading to marked functional limitations in affected individuals [1]. Currently, the prevalence of asthma is estimated at 12.6%, affecting more than 339 million people worldwide. Although asthma does not discriminate by age, race, or ethnicity, socioeconomic status and ethnic background have been shown to exert substantial effects on the prevalence, morbidity, and mortality of the disease in many countries. In addition, asthma constitutes a significant economic, social, and healthcare burden globally [2]. Asthma is characterized by symptoms such as wheezing, cough, shortness of breath, and chest tightness. These symptoms generally result from airway narrowing and inflammation triggered by environmental factors, allergens, or respiratory infections [3, 4]. Furthermore, symptoms such as low energy levels, fatigue, and excessive daytime sleepiness are frequently observed in patients with asthma. Fatigue, defined as a subjective feeling of exhaustion, is a significant daily symptom in asthma but is often overlooked. Studies indicate that severe fatigue is present in approximately 63% of outpatients with asthma [5].

In addition to pharmacological approaches, non-pharmacological methods play an important role in the management of asthma. Pharmacological treatment includes bronchodilators and inhaled corticosteroids, whereas non-pharmacological approaches aim to control symptoms, prevent exacerbations, and improve quality of life [1]. However, symptoms persist in approximately 5–10% of patients despite pharmacological treatment, highlighting the need for complementary non-pharmacological interventions [6]. In this context, breathing exercises are considered an important adjunctive method in asthma management due to their low cost, safety, and ease of application, and they can be implemented under nurse-led interventions [7, 8].

The buteyko breathing technique is a non-pharmacological breathing exercise approach used in the management of asthma and other respiratory diseases. Developed in the 1950s by the Ukrainian physiologist Konstantin Buteyko, this method represents a breathing retraining approach aimed at reducing hyperventilation and the associated hypocapnia [7, 9]. By restoring the balance between carbon dioxide (CO₂) and oxygen (O₂) at the cellular level, the technique seeks to improve breathing patterns and reduce the frequency and severity of asthma symptoms. The technique specifically focuses on breath-holding practices to regulate breathing patterns and to reduce hyperventilation and low carbon dioxide levels [10]. The buteyko breathing technique encompasses nasal breathing, diaphragmatic breathing, and breath-hold control. Nasal breathing facilitates the filtration, humidification, and warming of inspired air, while diaphragmatic breathing contributes to lung expansion by increasing intrapleural pressure [1, 10]. In addition, respiratory function is assessed using the Control Pause (CP) test, in which a CP duration of less than 10 s indicates dysfunctional breathing, whereas a duration exceeding 30 s reflects optimal breathing function [6].

Evidence regarding the effects of the Buteyko breathing technique on fatigue and functional exercise capacity remains limited. Moreover, further research is required to evaluate the feasibility of integrating this technique into nursing practice and its contribution to clinical outcomes. In this context, assessing the clinical effectiveness of the buteyko breathing technique in asthma management is of particular importance. Therefore, the aim of this study was to evaluate the effects of the Buteyko breathing technique applied to patients with asthma on fatigue levels, functional exercise capacity, and asthma control.

## Research hypotheses

### H0₁

The Buteyko breathing technique applied to patients with asthma has no effect on fatigue levels.

### H1

The Buteyko breathing technique applied to patients with asthma has an effect on fatigue levels.

### H0₂

The Buteyko breathing technique applied to patients with asthma has no effect on functional exercise capacity.

### H2

The Buteyko breathing technique applied to patients with asthma has an effect on functional exercise capacity.

### H0₃

The Buteyko breathing technique applied to patients with asthma has no effect on asthma control.

### H3

The Buteyko breathing technique applied to patients with asthma has an effect on asthma control.

## Materials and methods

### Study design

This study was planned as a pretest–posttest randomized controlled experimental trial to determine the effects of the Buteyko breathing technique on fatigue, exercise capacity, and asthma control in patients with asthma. The study was conducted between October 2024 and June 2025 among patients who presented to the outpatient clinic of the Department of Pulmonary Diseases at Gaziantep University Research and Application Hospital, located in the southern region of Türkiye.

### Study population and sample

**Fig. 1:**
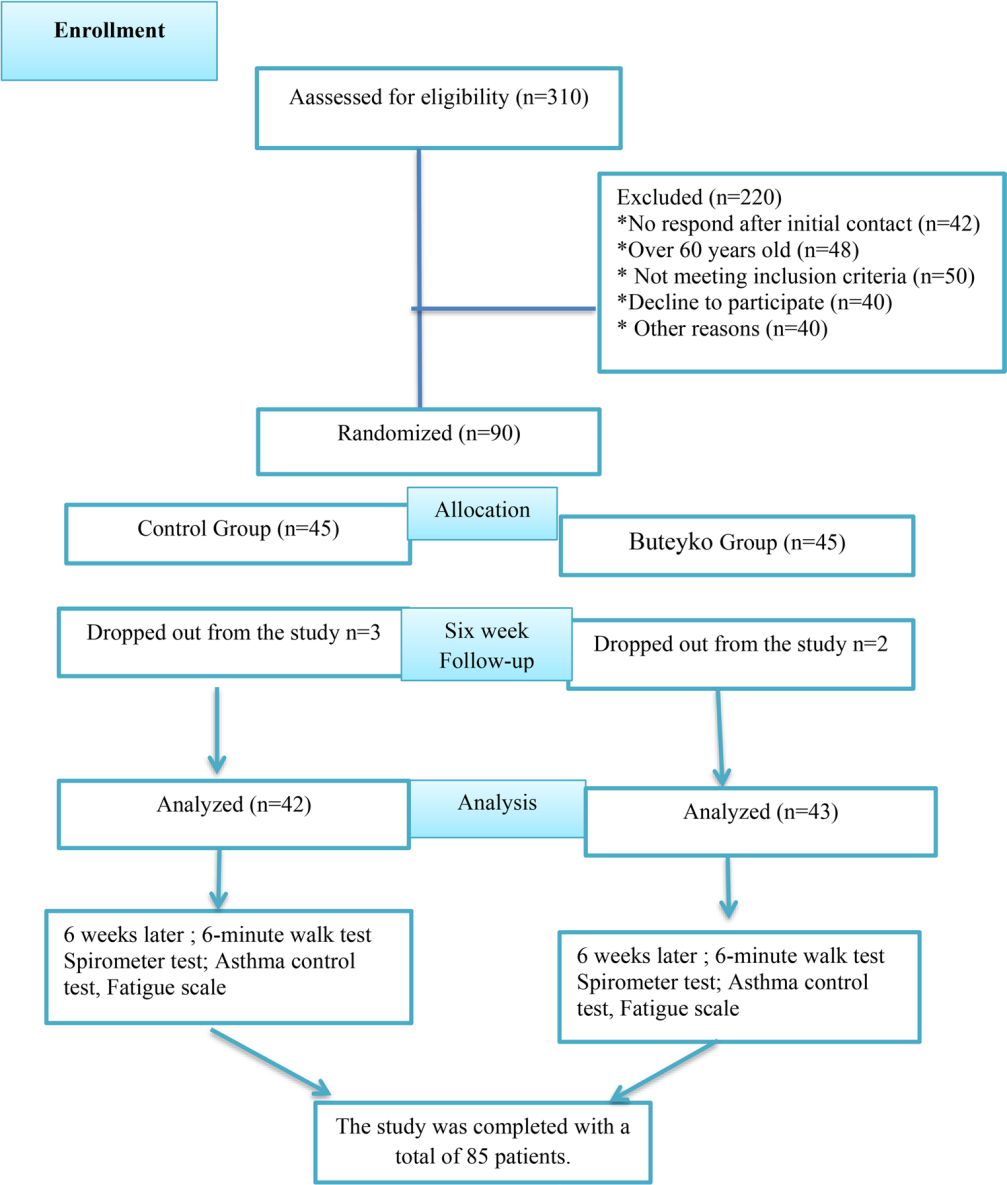
Sample selection flowchart according to CONSORT standards

The study population consisted of patients diagnosed with asthma who presented to the Pulmonary Diseases outpatient clinic of a university hospital between October 15, 2024, and June 25, 2025. The reporting of study outcomes was conducted in accordance with the CONSORT-Outcomes 2022 Extension guidelines [11] (Fig. 1). The clinical trial was registered (Clinical Trials.gov Identifier: NCT07353242). At the beginning of the study, 310 outpatients were contacted as potential participants. Of these individuals, 42 could not be reached, 48 did not meet the age criteria, 50 were excluded due to comorbid conditions, 40 declined to participate, and 40 were excluded because they were unable to attend the study for various reasons (e.g., time constraints, work-related issues, or being out of town). The final sample consisted of patients who met the inclusion criteria at the specified time and location and who voluntarily agreed to participate in the study. The sample size was calculated using the G*Power 3.1.9.7 software, taking into account sample sizes reported in previous asthma-related studies [7, 12, 13]. Based on an effect size of 0.30, a statistical power of 80%, and a significance level (α) of 0.05, the minimum required sample size was calculated as 80 participants. Considering a potential dropout rate of 10%, the total sample size was increased to 90 patients with asthma, with 45 participants allocated to the control group and 45 to the intervention group. A total of 90 participants were randomly assigned to either the control group (*n* = 45) or the intervention group (*n* = 45). During the study period, five participants withdrew, and the study was completed with a total of 85 participants.

### Inclusion and exclusion criteria

**Exclusion Criteria:**

- Presence of an acute exacerbation of asthma.
- Conditions that impair walking ability.
- Communication or cognitive impairments.
- Presence of an infectious disease other than asthma.

**Inclusion Criteria:**

- A confirmed diagnosis of asthma for at least the past 6 months.
- Absence of heart failure.
- No communication or cognitive impairments.
- No conditions limiting ambulation.
- Age between 18 and 59 years.

### Randomization and blinding

Participants were allocated to the intervention and control groups using a computer-based simple randomization method. Randomization was performed by an independent specialist who was not involved in the research process, using a computer-generated random number list. Block randomization was not applied; each participant who met the inclusion criteria was assigned to a study group according to the random allocation sequence generated by the computer program. Blinding was maintained as much as possible throughout the study. The researchers responsible for data collection and the researchers conducting the statistical analyses were blinded to group allocation (single-blind design). Data were recorded using group codes, and all analyses were performed without knowledge of group identity.

### Data collection tools

#### Information form

The information form consisted of a total of 23 items. Of these, seven questions addressed sociodemographic characteristics (e.g., age, sex, education), while the remaining 16 questions focused on disease-related characteristics (e.g., duration of asthma, family history of asthma, history of allergies, and number of asthma attacks).

#### Asthma control test (ACT)

The Asthma Control Test is a brief, clear, and easy-to-administer assessment tool that can be completed by patients or their relatives. The questionnaire consists of five items with five-point Likert-type response options. The total score ranges from a minimum of 5 to a maximum of 25. A score of 25 indicates complete asthma control, scores between 20 and 24 indicate partially controlled asthma, and scores of 19 or below indicate uncontrolled asthma. The Turkish validity and reliability study of the ACT was conducted by Uysal et al. 2012 [14].

### Chronic obstructive pulmonary disease and asthma fatigue scale (CAFS)

The Chronic Obstructive Pulmonary Disease and Asthma Fatigue Scale was developed to assess fatigue levels in individuals diagnosed with COPD and asthma. Originally developed by Revicki et al. in 2010, the Turkish validity and reliability study of the scale was conducted by Arslan and Öztunç in 2013. The scale consists of 12 items rated on a five-point Likert scale ranging from 1 (“never”) to 5 (“very often”); however, items 11 and 12 are reverse scored. Raw item scores are summed and converted to a scale score ranging from 0 to 100. The minimum possible total score is 12 and the maximum is 60, with higher scores indicating greater levels of fatigue. The Cronbach’s alpha coefficient of the original Turkish version was reported as 0.92 [15]. In the present study, the Cronbach’s alpha coefficient was calculated as 0.75.

#### Pulmonary function test (PFT)

Pulmonary function testing was performed in the hospital’s respiratory measurement laboratory using a standard spirometer by a trained technician. Within the scope of the test, respiratory parameters including forced expiratory volume in one second (FEV₁) and the FEV₁/forced vital capacity (FVC) ratio were evaluated and recorded in both liters and percentages of predicted values. Measurements were conducted twice for each patient: at baseline and at the end of the sixth week.

#### Six-minute walk test (6MWT)

The six-minute walk test, a widely used method for assessing exercise capacity in chronic respiratory diseases [16], was administered in accordance with international guidelines along a 20-meter flat corridor, and the total distance walked in six minutes was recorded. Participants were instructed to rest for 10 min prior to the test and to avoid heavy meals for at least 2 h before testing, as well as to wear appropriate clothing. Blood pressure, heart rate, oxygen saturation (SpO₂), respiratory rate, and perceived dyspnea were assessed before and after the test. During the test, SpO₂ was continuously monitored, and the test was terminated for safety reasons if SpO₂ dropped below 85%. After completion of the test, factors limiting performance and any potential adverse events were documented.

#### Buteyko group

The core component of the Buteyko method involves reducing hyperventilation through the combination of controlled breathing reduction periods, described as “slow and reduced breathing,” and breath-holding periods known as “controlled” and “extended pauses” [17]. In addition to the standard treatment protocol, participants in the intervention group received training and practice in the Buteyko Breathing Technique (BBT). The intervention was delivered by a researcher holding an international certification in the Buteyko method and was conducted as face-to-face group training in a quiet and calm room within the hospital’s outpatient clinic. Prior to the intervention, participants received a 30-minute educational session covering the purpose, fundamental principles, and practical application of the Buteyko breathing technique. Following the training, participants were seated comfortably in chairs, first performed normal breathing exercises, and subsequently engaged in the Buteyko breathing exercises, which were applied in two stages.

*The first stage was the Control Pause (CP);* The CP is a measure of how long an individual can comfortably hold their breath after a normal exhalation. When the CP is low, respiratory volume does not align with the body’s metabolic demands, which may lead to an increase in symptoms associated with hyperventilation. During this procedure, the mouth was gently taped with soft paper tape throughout the session to prevent mouth breathing. Participants were first instructed to breathe normally through the nose for 10 s. Following this preparatory exercise, they were asked to fully exhale and then hold their breath by pinching the nose with the thumb and index finger until a sensation of air hunger was perceived. Control pause durations were recorded during this period. Participants were instructed not to strain during breath holding and to release the nose and resume nasal breathing as soon as the urge to breathe occurred. This procedure was repeated six times in the seated position.

*Second stage breath-holding walking exercise;* The exercise began with participants standing upright. Initially, a few small breaths were taken and exhaled, followed by closing the nose and holding the breath. Walking was initiated during the breath-hold, which was continued only until the first involuntary urge to breathe arose, without intentionally prolonging the breath-hold. After this point, the nose was released, allowing normal nasal breathing to resume. If participants felt the need to take a deep breath or breathed through the mouth immediately after the walk, this indicated that the breath-hold duration had been excessively long. The exercise was repeated, gradually increasing the number of steps in a slow and controlled manner. Care was taken to ensure that the exercise remained mildly challenging without causing significant discomfort or distress. Participants were instructed to perform these exercises at home three times daily, following the same procedure. They were monitored weekly at the outpatient clinic, and adherence and technique were supervised throughout the six-week intervention period.

#### Control group

Participants in the control group continued with their routine medical treatment. At baseline, the six-minute walk test, spirometry, Asthma Control Test, and Fatigue Scale were administered. After six weeks, these participants were reassessed in the outpatient clinic using the same tests and scales. At the conclusion of the study, participants in the control group were provided training in the buteyko breathing exercises.

### Statistical analysis

Statistical analyses were performed using SPSS version 25.0 (IBM, New York, USA). Data were presented as mean ± standard deviation for continuous variables and as frequencies for categorical variables. The normality assumption was assessed using the Shapiro–Wilk test. The chi-square test was used to compare categorical variables. For comparisons between two independent groups, the independent samples t-test was applied, while one-way analysis of variance was used for comparisons among three or more groups. Relationships between variables were examined using correlation analysis. To evaluate the magnitude of differences between groups, Cohen’s d effect sizes with 95% confidence intervals were calculated. Statistical significance was set at *p* < 0.05.

### Ethical considerations

Following the development of the research protocol, ethical approval was obtained from the Non-Interventional Research Ethics Committee of XXX University, located in southeastern Turkey (Date: 31.07.2024/ Decision No: 2024/186). Additionally, all necessary institutional permissions from the healthcare facility where the study was conducted were obtained. Participants were provided with detailed information regarding the purpose and scope of the study, and written and verbal informed consent was obtained on a voluntary basis. Participants were informed that they could withdraw from the study at any stage without any consequences. The study was conducted in accordance with the ethical principles outlined in the Declaration of Helsinki.

## Results

The mean age of participants in the intervention group was 43.44 ± 11.11 years (range: 21–59), while the mean age of the control group was 42.78 ± 11.63 years (range:20–59). In the intervention group, 18.6% of participants were aged 20–29 years, 11.6% were 30–39 years, 27.9% were 40–49 years, and 41.9% were 50–59 years. In the control group, the corresponding age distribution was 16.7%, 16.7%, 31.0%, and 35.6%, respectively. No statistically significant difference was observed between the groups in terms of age distribution (*p* = 0.873). Regarding gender, 69.8% of the intervention group were female and 30.2% were male, while in the control group 66.7% were female and 33.3% were male. The difference in gender distribution between the groups was not statistically significant (*p* = 0.759). Educational status showed that in the intervention group, 14.0% were literate, 44.2% had completed primary school, 20.9% had completed high school, and 20.9% held a bachelor’s degree. In the control group, these rates were 11.9%, 26.2%, 40.5%, and 21.4%, respectively. No statistically significant difference was found between the groups regarding education level (*p* = 0.197) Table 1.

**Table 1:** Comparison of descriptive characteristics of asthma patients by group

Socio-demographic characteristics	Intervention (*n* = 43)		Control (*n* = 42)		Test	*p*
	*n*	%	*n*	%		
Age 20–29 30–39 40–49 50–59	8 5 12 18	18.6 11.6 27.9 41.9	7 7 13 15	16.7 16.7 31.0 35.6	X^2^=0.886	0.873
Min-Max Mean ± SD (age)	21.0–59.0 43.44 ± 11.11		20.0–59.00 42.78 ± 11.63		X^2^=0.340	0.348
Gender Female Male	30 13	69.8 30.2	28 14	66.7 33.3	X^2^=0.470	0.759
Education Literacy Primary Education High School Undergraduate	6 19 9 9	14.0 44.2 20.9 20.9	5 11 17 9	11.9 26.2 40.5 21.4	X^2^=0.206	0.197
Marital Status Married Single	35 8	81.4 18.6	33 9	78.6 21.4	X^2^=0.792	0.745
Occupation Housewife Worker Civil servant Retired Self-employed	25 2 5 3 8	58.1 4.7 11.6 7.0 18.6	19 5 4 3 11	45.2 11.9 9.5 7.1 26.2	X^2^=0.618	0.613
Income Good Medium Bad	3 34 6	7.0 79.0 14.0	2 29 11	4.8 69.0 26.2	X^2^=0.408	0.358

X^2=^ Chi-square test based on the exact method, *p*<0.05

In terms of marital status, 81.4% of the intervention group were married and 18.6% were single, compared to 78.6% married and 21.4% single in the control group (*p* = 0.745). Occupational distribution in the intervention group was 58.1% housewives, 4.7% laborers, 11.6% civil servants, 7.0% retirees, and 18.6% self-employed. In the control group, the distribution was 45.2%, 11.9%, 9.5%, 7.1%, and 26.2%, respectively. No significant difference was observed between the groups regarding occupation (*p* = 0.613). Regarding income level, 79.0% of participants in the intervention group and 69.0% in the control group reported a moderate income. There was no statistically significant difference between the groups in terms of income level (*p* = 0.358) Table 2.

**Table 2:** Comparison of clinical characteristics of asthma patients according to groups

Clinical data	Intervention (*n* = 43)		Kontrol (*n* = 42)		Test	*p*
	*n*	%	*n*	%		
Family history of asthma Yes No	23 20	53.5 46.5	13 29	31.0 69.0	X^2^=0.048	**0.036**
Disease duration Mean ± SD (years)	11,53 ± 10.48		8.61 ± 5.73		t = 1.585	**0.005**
Allergy history Yes No	20 23	46.5 53.5	17 25	40.5 59.5	X^2^=0.663	0.575
Factors that worsen asthma: House dust Pollen Cold air and humidity Odors Cigarette smoke	9 6 11 7 10	20.9 14.0 25.6 16.2 23.3	9 6 14 6 7	21.4 14.3 33.3 14.3 16.7	X^2^=0.928	0.917
Additional chronic disease history Yes No	15 28	34.9 65.1	18 24	42.9 57.1	X^2^=0.509	0.451
Hospitalization in the last year Yes No	20 23	46.5 53.5	28 14	66.7 33.3	X^2^=0.081	0.061
Emergency room visit due to attack Yes No	33 10	76.7 23.3	33 9	78.6 21.4	X^2^=1.000	0.840
Number of attacks per year Mean ± SD (years)	2.18 ± 2.10		1.50 ± 1.17		t = 1.848	**0.022**
Smoking Yes No	19 24	44.2 55.8	24 18	57.1 42.9	X^2^=0.281	0.232
Year of smoking Mean ± SD (years)	18.29 ± 9.08		16.12 ± 8.03		t = 0.816	0.419
Using herbal remedies for asthma Yes No	11 32	25.6 74.4	14 28	33.3 66.7	X^2^=0.482	0.433

X^2=^ Chi-square test based on the exact method, *p*<0.05

When clinical characteristics were evaluated, the proportion of participants with a family history of asthma was 53.5% in the intervention group and 31.0% in the control group, with this difference being statistically significant (*p* = 0.036). The mean disease duration was 11.53 ± 10.48 years in the intervention group and 8.61 ± 5.73 years in the control group, indicating a significant difference between the groups (*p* = 0.005). A history of allergy was reported in 46.5% of the intervention group and 40.5% of the control group, with no statistically significant difference observed (*p* = 0.575). Among the triggers reported to exacerbate asthma, the most common in the intervention group were cold air and humidity (25.6%), cigarette smoke (23.3%), and household dust (20.9%). In the control group, the most frequent triggers were cold air and humidity (33.3%), household dust (21.4%), and cigarette smoke (16.7%), with no significant difference between the groups (*p* = 0.917).

The presence of additional chronic diseases was 34.9% in the intervention group and 42.9% in the control group (*p* = 0.451). Hospitalization within the past year was reported by 46.5% of the intervention group and 66.7% of the control group (*p* = 0.061). The mean annual number of asthma attacks was 2.18 ± 2.10 in the intervention group and 1.50 ± 1.17 in the control group, with the difference being statistically significant (*p* = 0.022). Emergency department visits due to asthma attacks were reported in 76.7% of the intervention group and 78.6% of the control group, with no significant difference observed (*p* = 0.840).

Regarding smoking status, 44.2% of participants in the intervention group and 57.1% in the control group were smokers (*p* = 0.232). The mean duration of smoking was 18.29 ± 9.08 years in the intervention group and 16.12 ± 8.03 years in the control group, with no significant difference (*p* = 0.419). The use of herbal remedies for asthma was reported by 25.6% of the intervention group and 33.3% of the control group, with no significant difference observed (*p* = 0.433) Table 3.

**Table 3:** Comparison of mean scores of buteyko breathing exercise application on fatigue, exercise capacity, and asthma control in asthma patients by group

Measuring instruments	Intervention (*n* = 43)	Control (*n* = 42)	Test	*p*	Cohen’s
6 min walking (meters) pre-test post-test	445.93 ± 73.09 498.83 ± 71.38	457.73 ± 64.99 461.54 ± 65.31	t=-0.786 t = 2.624	*p* = 0.434 *p* = 0.010	0.71
Expected walking percentage pre-test post-test	63.51 ± 11.69 70.83 ± 10.42	65.80 ± 9.25 66.46 ± 9.29	t=-1.003 t = 2.037	*p* = 0.319 *p* = 0.045	0.63
FVC (litre) pre-test post-test	3.01 ± 0.89 3.12 ± 0.75	3.30 ± 0.94 3.35 ± 0.89	t=-1.429 t=-1.262	*p* = 0.157 *p* = 0.211	0.07
FEV1 (litre) pre-test post-test	2.14 ± 0.76 2.53 ± 0.73	2.47 ± 0.75 2.48 ± 0.76	t=-2.020 t = 0.263	*p* = 0.047 *p* = 0.793	0.51
FVC/FEV1 (%) pre-test post-test	70.41 ± 11.64 74.13 ± 9.86	75.16 ± 9.69 74.73 ± 8.97	t=-2.046 t=-0.293	*p* = 0.044 *p* = 0.770	0.39
Fatigue Scale Total pre-test post-test	40.53 ± 4.08 33.00 ± 4.18	38.47 ± 5.95 38.16 ± 4.98	t=-1.863 t=-5.181	*p* = 0.066 p = **0.001**	1.42
ACT pre-test post-test	14.37 ± 3.80 20.20 ± 1.95	14.95 ± 3.09 15.14 ± 3.54	t=-0.770 t = 6.567	*p* = 0.444 p = **0.001**	1.34

t= Independent samples t test, *p*<0.05, Cohen’s d values interpreted as: 0.2 = small, 0.5 = medium, 0.8 = large

According to the six-minute walk test results, the walking distance in the intervention group increased from 445.93 ± 73.09 m at baseline to 498.83 ± 71.38 m at the post-test, which was statistically significant (t = 2.624; *p* = 0.010). The magnitude of this change was moderate to high (cohen’s d = 0.71). In the control group, the walking distance increased from 457.73 ± 64.99 m to 461.54 ± 65.31 m; however, this change was not statistically significant (t = − 0.786; *p* = 0.434).

The percentage of predicted walking distance increased in the intervention group from 63.51 ± 11.69% to 70.83 ± 10.42%, which was statistically significant (t = 2.037; *p* = 0.045) and represented a moderate to high effect size (cohen’s d = 0.63). In the control group, the predicted walking percentage increased from 65.80 ± 9.25% to 66.46 ± 9.29%, but the change was not statistically significant (t = − 1.003; *p* = 0.319).

In pulmonary function tests, forced vital capacity (FVC) increased from 3.01 ± 0.89 L to 3.12 ± 0.75 L in the intervention group, and from 3.30 ± 0.94 L to 3.35 ± 0.89 L in the control group; however, these changes were not statistically significant in either group (*p* > 0.05; cohen’s d = 0.07). Forced expiratory volume in one second (FEV₁) increased from 2.14 ± 0.76 L to 2.53 ± 0.73 L in the intervention group, which was statistically significant (t = − 2.020; *p* = 0.047) and showed a moderate effect size (cohen’s d = 0.51). In the control group, FEV₁ changed from 2.47 ± 0.75 L to 2.48 ± 0.76 L, showing no significant difference (*p* = 0.793). The FEV₁/FVC ratio increased from 70.41 ± 11.64% to 74.13 ± 9.86% in the intervention group, which was statistically significant (*p* = 0.044) with a small to moderate effect size (cohen’s d = 0.39). No significant change was observed in the control group (75.16 ± 9.69% to 74.73 ± 8.97%; *p* = 0.770).

The total score of the Fatigue Scale significantly decreased in the intervention group, from 40.53 ± 4.08 at baseline to 33.00 ± 4.18 at post-intervention (t = − 5.181; *p* = 0.001), indicating a very large effect size (cohen’s d = 1.42). In the control group, the total fatigue score decreased slightly from 38.47 ± 5.95 to 38.16 ± 4.98, but this change was not statistically significant (*p* = 0.066).

The Asthma Control Test score increased in the intervention group from 14.37 ± 3.80 at baseline to 20.20 ± 1.95 after the intervention (t = 6.567; *p* = 0.001), demonstrating a large effect size (cohen’s d = 1.34). In the control group, the ACT score increased slightly from 14.95 ± 3.09 to 15.14 ± 3.54, without a statistically significant difference (*p* = 0.444) Table 4.

**Table 4:** Regression analysis results regarding the effect of buteyko breathing exercise application on fatigue, exercise capacity, and asthma control in asthma patients according to groups

Variables	B	S.Error	β	t	*p*	95% CI Lower-Upper
Fixed	2.291	0.251		9.125	0.001	1.792 − 2.791
6 min walking pre-test post-test	0.012 -0.013	0.001 0.001	1.586 -1.687	10.434 -11.099	0.001 0.001	0.009 ─ 0.014 -0.015− -0.010
*R* =0.777. *R* ^2^ = 0.603. *F* = 62.354 p *=* 0.001						
Fixed	2.217	0.270		8.211	0.001	1.680 − 2.750
Expected walking percentage pre-test post-test	0.071 -0.078	0.008 0.009	1.496 -1.553	8.662 -8.994	0.001 0.001	0.055 ─ 0.088 -0.095− -0.060
*R* =0.709. *R* ^2^ = 0.503 *F* = 41.437 p *=* 0.001						
Fixed	1.210	0.221		5.473	0.001	0.770─1.650
FEC (liters) ön test son test	0.070 0.019	0.103 0.114	0.129 0.032	0.682 0.171	0.497 0.864	-0.134 − 0.275 -0.207─ 0.246
*R* =0.156. *R* ^2^ = 0.024. *F* = 1.023 p *=* 0.364						
Fixed	1.570	0.163		6.611	0.001	1.245 − 1.895
FEV1 (liters) pre-test post-test	0.803 -0.768	0.136 0.141	1.234 -1.135	5.926 -5.446	0.001 0.001	533─1.073 -1.049− -0.488
*R* =0.548 *R* ^2^ = 0.300 *F* = 17.572 p *=* 0.001						
Fixed	1.848	0.392		4.710	0.001	1.068─2.629
FEC/FEV1 (%) pre-test post-test	0.063 -0.066	0.012 0.014	1.367 -1.237	5.354 -4.843	0.001 0.001	0.040 − 0.086 -0.094─ -0.039
*R* =0.510 *R* ^2^ = 0.260 *F* = 14.389 p *=* 0.001						
Fixed	1.083	0.331		3.277	0.002	0.426─1.741
Fatigue Scale Total pre-test post-test	-0.057 0.075	0.009 0.009	-0.585 0.782	-6.543 8.749	0.001 0.001	-0.074− -0.040 0.058 ─ 0.092
*R* =0.710 *R* ^2^ = 0.504 *F* = 41.590 p *=* 0.001						
Fixed	2.511	0.229		10.977	0.001	2.056 − 2.966
ACT pre-test post-test	0.054 -0.105	0.013 0.013	0.373 -0.732	4.221 -8.282	0.001 0.001	0.029**─**0.080 -0.131− -0.080
*R* =0.678 *R* ^2^ = 0.459 *F* = 34.837 p *=* 0.001						

*CI* Confidence Interval

The regression model for the six-minute walk test (6MWT) distance was statistically significant (*R* = 0.777; R²=0.603; F = 62.354; *p* = 0.001), explaining 60.3% of the total variance in 6MWT distance. Both the baseline (B = 0.012; *p* = 0.001) and post-intervention (B = − 0.013; *p* = 0.001) variables had significant effects on the dependent variable.

The regression model for the predicted walking percentage was also statistically significant (*R* = 0.709; R²=0.503; F = 41.437; *p* = 0.001), accounting for 50.3% of the total variance. Both the baseline (B = 0.071; *p* = 0.001) and post-intervention (B = − 0.078; *p* = 0.001) variables significantly predicted the dependent variable.

The regression model for FVC was not statistically significant (*R* = 0.156; R²=0.024; F = 1.023; *p* = 0.364), explaining only 2.4% of the variance. Neither baseline (*p* = 0.497) nor post-intervention (*p* = 0.864) variables had significant effects. The regression model for FEV₁ was statistically significant (*R* = 0.548; R²=0.300; F = 17.572; *p* = 0.001), accounting for 30.0% of the total variance. Both baseline (B = 0.803; *p* = 0.001) and post-intervention (B = − 0.768; *p* = 0.001) variables significantly affected the dependent variable.

The regression model for the FEV₁/FVC ratio was significant (*R* = 0.510; R²=0.260; F = 14.389; *p* = 0.001), explaining 26.0% of the variance. Both baseline (B = 0.063; *p* = 0.001) and post-intervention (B = − 0.066; *p* = 0.001) variables contributed significantly to the model.

The regression model for the total Fatigue Scale score was statistically significant (*R* = 0.710; R²=0.504; F = 41.590; *p* = 0.001), explaining 50.4% of the total variance in fatigue levels. Both baseline (B = − 0.057; *p* = 0.001) and post-intervention (B = 0.075; *p* = 0.001) variables were identified as significant predictors.

The regression model for the Asthma Control Test score was also statistically significant (*R* = 0.678; R²=0.459; F = 34.837; *p* = 0.001), explaining 45.9% of the total variance in asthma control. Both baseline (B = 0.054; *p* = 0.001) and post-intervention (B = − 0.105; *p* = 0.001) variables had significant effects on the dependent variable.

## Discussion

Breathing exercises are widely used worldwide as a non-pharmacological treatment approach for individuals with asthma. These interventions aim to improve asthma symptom control and may be implemented in various forms, including diaphragmatic breathing techniques, the buteyko breathing method, the Papworth method, yogic breathing, or other interventions designed to optimize breathing patterns [18]. In the present study, the application of the buteyko breathing technique resulted in statistically significant and clinically meaningful improvements in functional capacity, pulmonary function, fatigue levels, and asthma control among individuals with asthma in the intervention group. In contrast, the absence of substantial changes in these parameters in the control group supports the interpretation that the observed improvements were attributable specifically to the intervention. Furthermore, the lack of significant differences between groups in terms of sociodemographic characteristics reduces the potential influence of confounding variables, thereby strengthening the internal validity of the findings and supporting the conclusion that the observed effects are primarily associated with the implemented intervention.

The walking test is used to assess exercise tolerance, suitability for rehabilitation, and the appropriate workload applied during exercise. It is also employed to evaluate the effectiveness of recovery. The six-minute walk test provides a simple and practical method for assessing exercise tolerance, adaptation to activities of daily living, treatment effectiveness, and prognosis. It is widely used in clinical practice to measure exercise capacity [16]. In a study conducted in Belgium, the mean 6MWT distance among healthy adults aged 18–80 years was reported as 625 ± 90.7 m [19].

In the present study, analysis of the six-minute walk test results demonstrated a statistically significant increase in walking distance from pre-test to post-test in the intervention group (*p* = 0.010), with a moderate-to-high effect size. In contrast, no statistically significant change in walking distance was observed in the control group. The percentage of predicted walking distance increased significantly in the intervention group (from 63.51% to 70.83%; *p* = 0.045; cohen’s d = 0.63), whereas no significant change was detected in the control group. These findings indicate that the applied intervention was effective in improving functional exercise capacity in individuals with asthma. Similarly, systematic reviews and meta-analyses conducted by Feng et al. (2021) and Osandik et al. (2022), which examined the effects of exercise-based pulmonary rehabilitation in adults with asthma, reported significant increases in 6MWT distance in intervention groups compared with control groups [20, 21]. These findings are consistent with the 6MWT results observed in the present study.

In this study, respiratory function tests were examined following the application of buteyko breathing exercises in individuals with asthma. Although FVC values showed an increasing trend in both the intervention and control groups, this change was not statistically significant (*p* > 0.05; cohen’s d = 0.07). In contrast, forced expiratory volume in one second (FEV₁) increased significantly in the intervention group (*p* = 0.047; cohen’s d = 0.51), while no significant change was observed in the control group. Similarly, the FEV₁/FVC ratio increased significantly in the intervention group (*p* = 0.044; cohen’s d = 0.39), whereas it remained unchanged in the control group.

These findings are consistent with the study by Grznár et al. (2022), in which breathing exercises and a combination of breathing plus aerobic exercises in individuals with asthma resulted in significant improvements in FEV₁ and the FEV₁/FVC ratio, while changes in FVC were limited [17]. This pattern suggests that breathing exercises may exert more pronounced positive effects on airflow-related parameters, whereas their impact on vital capacity appears to be relatively modest. Similarly, Beyece İncazlı et al. (2025) reported that the buteyko breathing technique, as a complementary approach in asthma management, produced favorable effects on pulmonary function tests, with post-intervention improvements observed in FEV₁ and the FEV₁/FVC ratio [22]. In addition, Koyuncu et al. (2025) demonstrated a significant increase in the FEV₁/FVC ratio following the implementation of breathing exercises in individuals with asthma [23].

Conversely, the study by Vagedes et al. (2024) reported that while the Buteyko breathing technique improved clinical symptoms, no overall significant changes were observed in spirometric parameters (FEV₁, FVC, FEV₁/FVC) [13]. This comparison supports the notion that although breathing exercises may improve airway function in some patients, their effects can vary depending on population characteristics, exercise modality, and duration of intervention. The observed improvements in FEV₁ and the FEV₁/FVC ratio in the present study suggest that the applied exercise protocol and patient adherence may have contributed positively to these parameters. In conclusion, these findings indicate that buteyko breathing exercises may improve airflow-related parameters in asthma, leading to clinically meaningful changes particularly in FEV₁ and the FEV₁/FVC ratio; however, their effect on volumetric indices such as FVC appears to be limited. This underscores the importance of evaluating breathing exercises not only through spirometric outcomes but also in conjunction with non-spirometric clinical outcomes, including symptom scores and quality of life measures.

In the present study, following the implementation of the buteyko breathing exercise, the mean Asthma Control Test score in the intervention group was significantly higher and reached statistical significance (*p* = 0.001), with a large effect size (cohen’s d = 1.34). In contrast, no statistically significant change in mean ACT scores was observed in the control group (*p* = 0.444). These findings indicate that buteyko breathing exercises produced a clinically meaningful improvement in asthma control in the intervention group.

The regression analysis examining asthma control was also statistically significant, with the model explaining approximately 45.9% of the total variance in asthma control levels (*R* = 0.678; R²=0.459; F = 34.837; *p* = 0.001). Within the model, both pre-test (B = 0.054; *p* = 0.001) and post-test (B = − 0.105; *p* = 0.001) variables were found to have significant effects on the dependent variable. These results demonstrate that both baseline and post-intervention measurements exert clinically meaningful effects on asthma control and that breathing exercises account for a substantial proportion of the variance in asthma control outcomes.

These findings are consistent with recent studies in the literature. In a randomized controlled trial conducted by Çelik and Yürük (2025), significant improvements in asthma control scores were observed in children aged 7–12 years with asthma who underwent the Buteyko breathing technique, with post-test scores significantly higher than pre-test values (*p* < 0.05) [12]. Similarly, in a randomized controlled study by Beyece İncazlı et al. (2025), the buteyko breathing technique, applied as a complementary approach to asthma treatment, demonstrated positive effects on asthma control, with significant improvements in ACT scores observed in the intervention group following the Buteyko training [22]. Furthermore, Hasoon and Abdulwahhab (2024) reported that a program incorporating the buteyko technique, pursed-lip breathing, and inhaler education significantly improved asthma control, highlighting the effectiveness of multidisciplinary respiratory education programs in reducing asthma symptoms and improving control scores [8]. Additionally, Elkafrawy et al. (2025) demonstrated that buteyko breathing exercises significantly enhanced asthma control in elderly patients with asthma, with marked increases in ACT scores observed following the intervention [7]. Taken together, the large effect size and regression model findings observed in the present study strongly support the robust impact of buteyko breathing exercises on asthma control. Moreover, the regression analysis indicates that both baseline asthma control levels and post-intervention changes contribute significantly to asthma control outcomes. These results suggest that breathing exercises can be effectively incorporated as a complementary strategy in asthma management across both pediatric and adult populations.

In the present study, following the implementation of the buteyko breathing exercise, the Total Fatigue Scale score in the intervention group decreased significantly (*p* = 0.001), with a very large effect size (cohen’s d = 1.42). In contrast, a slight reduction in TFS scores was observed in the control group; however, this change did not reach statistical significance (*p* = 0.066). These findings indicate that intervention-specific breathing exercises can markedly reduce subjective fatigue levels in individuals with asthma.

The regression model constructed for fatigue outcomes was also statistically significant and explained 50.4% of the total variance in fatigue levels (*R* = 0.710; R²=0.504; F = 41.590; *p* = 0.001). Within the model, both pre-test (B = − 0.057; *p* = 0.001) and post-test (B = 0.075; *p* = 0.001) variables were identified as significant predictors of fatigue. This finding suggests that the intervention exerted a strong and comprehensive effect on fatigue, and that changes between measurement points accounted for a substantial proportion of fatigue variance.

Fatigue is widely recognized in the literature as a prevalent and clinically significant complaint among individuals with asthma. Epidemiological studies have demonstrated that more than 60% of patients with asthma experience severe fatigue, which is closely associated with asthma control, quality of life, and dyspnea [5]. Moreover, fatigue has been identified as one of the most frequently reported and functionally limiting symptoms in individuals with severe asthma, yet it is often insufficiently addressed in standard clinical assessments [24].

One of the limited studies directly examining the effects of breathing exercises on fatigue in individuals with asthma, conducted by Ibrahim Abd El Kader et al. (2023), applied alternate nostril breathing and diaphragmatic breathing exercises and reported significant reductions in fatigue scores with both techniques (*p* < 0.001), with diaphragmatic breathing demonstrating a more pronounced effect. These findings highlight the effectiveness of breath control in reducing asthma symptoms and subjective burden such as fatigue [25]. Consistent with this evidence, the present findings support the notion that the Buteyko breathing technique may reduce fatigue through similar physiological mechanisms.

The beneficial effects of breathing- and relaxation-based interventions on fatigue have also been demonstrated in other chronic respiratory diseases. Neşe and Bağlama (2022) reported that progressive muscle relaxation and deep breathing exercises significantly reduced fatigue symptoms in patients with chronic obstructive pulmonary disease. Although the patient populations differ, this evidence suggests that breathing-focused behavioral interventions may have the potential to alleviate fatigue, a common symptom across chronic respiratory conditions [26] .

The most recent and robust evidence regarding the effects of exercise-based approaches on fatigue was presented in a systematic review by Burge et al. (2024), which demonstrated that graded exercise therapy significantly and consistently reduced fatigue in individuals with severe respiratory disease. This review clearly identifies fatigue as a modifiable clinical target in respiratory conditions [27] and supports the very large effect size observed following the Buteyko breathing intervention in the present study.

Furthermore, the regression model explaining 50.4% of the total variance in fatigue scores suggests that breathing exercises may serve as a strong and independent predictor of fatigue reduction in individuals with asthma.

### Clinical implications for nursing practice

This study demonstrates that buteyko breathing exercises can be integrated into nursing care as a complementary intervention for individuals with asthma. Nurses can support asthma control and symptom management by incorporating breathing exercise education into patient education programs. The observed improvements in asthma control, functional exercise capacity, and fatigue indicate that breathing exercises represent a feasible and effective component of nurse-led care. These findings support the inclusion of breathing exercises in clinical practice as a low-cost, safe, and non-pharmacological nursing intervention.

## Conclusions

This study demonstrated that buteyko breathing exercises increased functional exercise capacity in individuals with asthma and led to significant improvements in pulmonary function, particularly in FEV₁ and the FEV₁/FVC ratio. While asthma control markedly improved in the intervention group, a clinically meaningful reduction with a large effect size was observed in fatigue levels. In contrast, no significant changes were detected in these parameters in the control group. Overall, these findings support the effectiveness of buteyko breathing exercises as a complementary approach in asthma management.

### Recommendations

It is recommended that buteyko breathing exercises be incorporated as a complementary approach into routine treatment programs for individuals with asthma. Future studies should evaluate long-term follow-up outcomes and the effects of breathing exercises on quality of life using larger sample sizes. Additionally, randomized controlled trials examining the effectiveness of breathing exercises across different asthma severity levels and age groups are warranted.

### Study limitations

This study has several limitations that should be acknowledged. First, the relatively small sample size may limit the generalizability of the findings. Second, the relatively short study duration (6 weeks) did not allow for the evaluation of the long-term effects of buteyko breathing exercises. In addition, the assessment of certain variables, such as fatigue and asthma control, using self-reported measures may have introduced response bias. The use of a single-center study design and the absence of intervention delivery by multiple practitioners represent additional factors that may restrict the external validity of the results. Therefore, these limitations should be considered when interpreting the findings.

### Strengths of the study

One of the major strengths of this study is its comprehensive evaluation of the multidimensional effects of buteyko breathing exercises in individuals with asthma. Both objective and subjective outcomes, including functional exercise capacity, pulmonary function, asthma control, and fatigue, were assessed concurrently. The absence of significant differences in sociodemographic characteristics between the intervention and control groups strengthens the attribution of the observed effects to the intervention. The use of standardized instruments and assessments conducted by a trained technician reduced measurement error and enhanced the internal validity of the study. Furthermore, the prior validation and reliability of the assessment scales in the Turkish language represent an important strength, supporting the scientific robustness of the findings.

## Data Availability

The datasets used and analyzed during the study are available from the corresponding author on reasonable request.
